# Axial X-Ray Microscopy in Nanotomography

**DOI:** 10.3390/tomography12030041

**Published:** 2026-03-18

**Authors:** Konstantin P. Gaikovich, Ilya V. Malyshev, Dmitry G. Reunov, Nikolay I. Chkhalo

**Affiliations:** Institute for Physics of Microstructures, Russian Academy of Sciences, 603950 Nizhny Novgorod, Russia; ilya-malyshev@ipmras.ru (I.V.M.); reunov_dima@ipmras.ru (D.G.R.); chkhalo@ipmras.ru (N.I.C.)

**Keywords:** soft X-ray microscopy, EUV microscopy, tomography, absorption coefficient, numerical simulation, cell tomography

## Abstract

Background/Objectives: This article develops theory and methods for 3D tomographic imaging of absorption coefficient distributions using axial scanning with EUV microscopes at 46× and 345× magnification. Unlike conventional CT that requires sample rotation, axial scanning moves cells through the microscope focus. The aim is tomographic reconstruction of living cell fine structure without the organelle staining used in optical fluorescence microscopy or ultra-thin cell slicing as in electron microscopy. Methods: By generalizing the geometric-optical approximation for small absorption coefficient inhomogeneities in absorbing media, we derived a new explicit tomography equation and solution algorithm validated through numerical simulation. The approach was applied to *Convallaria* cell analysis using the ×46 microscope. For the ×345 microscope, we developed an alternative method where the kernel of the tomography integral equation was determined experimentally using gold nanospheres with known absorption coefficient, shape, and position. This method was tested through modeling and applied to diagnostics of *Convallaria* and mouse cerebellar granule cells. Results: The developed methods resolve subcellular features down to 140 nm using the ×46 microscope and 50 nm using the ×345 microscope. Thin low-contrast intracellular structures and individual 50–100 nm organelles were detected. Conclusions: Methods for retrieving absorption coefficient distributions in cone-beam geometry based on geometric-optical theory generalization and on calibration by gold nanoparticles have been developed and validated through numerical simulation and cell analysis. These methods demonstrate for the first time the effectiveness of axial nanotomography using multilayer mirror microscopes for cell diagnostics.

## Simple Summary

We developed a new tomography method for living cells using a special X-ray microscope. Our method is free from limitations related to the complicated preparation steps needed in other techniques—no staining of selected cell organelles (as in optical microscopy) and no slicing cells into ultra-thin sections (as in electron microscopy). Here is how it works: We scan the cell point by point with a focused X-ray beam. The signal from the focused spot is much stronger than from surrounding areas. This allows us to use mathematical techniques to reconstruct detailed images of the cell’s tiniest structures with the clarity we need.

## 1. Introduction

Soft X-ray microscopy has been developing for over 30 years, operating in the “water transparency window” spectral range (wavelengths 2.3–4.4 nm) [1,2,3,4] and at 13.4–13.8 nm wavelengths (EUV microscopy) [5,6,7]. The primary advantages of X-ray microscopy in the water window include nanometer-scale spatial resolution enabled by short wavelengths and high absorption contrast between carbon-rich structures. This permits examination of samples without contrasting or fluorescent substances. The relatively high radiation transmission and negligible scattering enable examination of cells and tissue samples in natural and potentially living states.

Optical microscopy operating in visible light does not permit direct investigation of living-cell nanostructure at such high resolutions [8]. Resolution enhancement methods in optical microscopy, such as STED microscopy [9], can significantly exceed the diffraction limit, achieving a tens of nanometers resolution. However, as with classical fluorescence microscopy, these methods visualize only stained organelles isolated from their biological environment, complicating the understanding of intracellular processes. Transmission electron microscopy achieves nanometer spatial resolution [10] but requires sample freezing and slicing into 100–500 nm films [11]. This destructive method precludes whole-cell studies. Atomic force and scanning tunneling microscopy examine only sample surfaces.

We are developing the theory and methods of X-ray axial tomography. Currently, full-mirror EUV microscopes with 13.84 nm wavelength are being developed. In this technique, samples are mounted on piezoelectric actuators and shifted along the microscope optical axis while recording X-ray image series as sample sections at different depths, which are sequentially positioned in the lens focal plane.

For microtomography based on the previously developed ×46 microscope, we propose and apply deconvolution methods for three-dimensional (3D) convolution-type integral equations. In [12], the calculated probing field distribution (incorporating measured mirror objective aberrations) served as the equation kernel. Subsequently, in [13,14], an inverse tomography problem integral equation was explicitly obtained based on Radon transformation formalism in the low-absorption geometric-optical approximation. The solution algorithm was investigated through numerical simulation [13] and applied to plant-cell analysis [14]. Tomographic reconstruction of relatively transparent cell regions demonstrated the algorithm’s ability to resolve fine structural details down to the microscope’s diffraction-limited resolution of 140 nm. However, in significant regions of these cells, the small absorption approximation was not satisfied, which complicated their analysis and accurate tomogram calibration.

In this work, we have obtained a significant generalization of the geometric-optical method theory—the tomography equation for small absorption coefficient inhomogeneities in absorbing media has been derived. A solution algorithm has been developed and validated through numerical simulations and tomographic analysis of *Convallaria* cells based on ×46 microscope measurements. This enabled justified application of this method throughout the entire cell volume and investigation of inhomogeneities in regions with elevated absorption, where small inhomogeneities undetectable by the previous algorithm were successfully identified. An equally important result was the tomogram calibration method proposed within this approach for more accurate quantitative analysis.

A novel tomography method has been developed for the ×345 microscope. The 3D convolution equation kernel was determined experimentally using test objects with a known absorption coefficient, geometry, and position. Algorithm testing results in numerical simulation and application to plant cells and mouse cerebellar granule cell neurites demonstrate 50 nm resolution in reconstructed tomographic images of individual cell fragments.

## 2. Materials and Methods

### 2.1. Instrumentation

**Figure 1:**
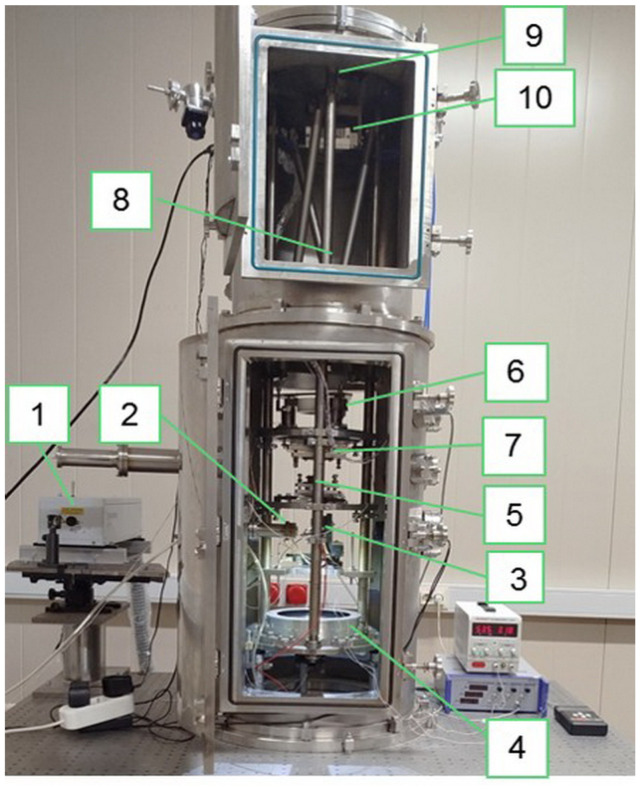
Photo of ×345 EUV microscope: 1—IR Nd:YAG laser (λ = 1064 nm); 2—beam-focusing lens; 3—gas valve with a conical nozzle (Ar); 4—elliptical multilayer Mo/Si mirror-collector, 5—test sample placed on a piezo actuator with the ability to shift along the *z*-axis; 6—multilayer Mo/Si aspherical concave mirror (magnification of two mirrors ×46); 7—multilayer Mo/Si convex mirror; 8—multilayer Mo/ZrSi2 filter; 9—convex mirror ×7.5; 10—detector (BSI CMOS, matrix 13.3 mm × 13.3 mm with 6.5 μm pixels).

Figure 1 shows the EUV microscope with 345-fold magnification. Its principal feature compared to the ×46 EUV microscope is an additional mirror providing 7.5 times additional magnification. Both microscopes have been developed at IPM RAS (Nizhny Novgorod, Russian Federation).

**Figure 2:**
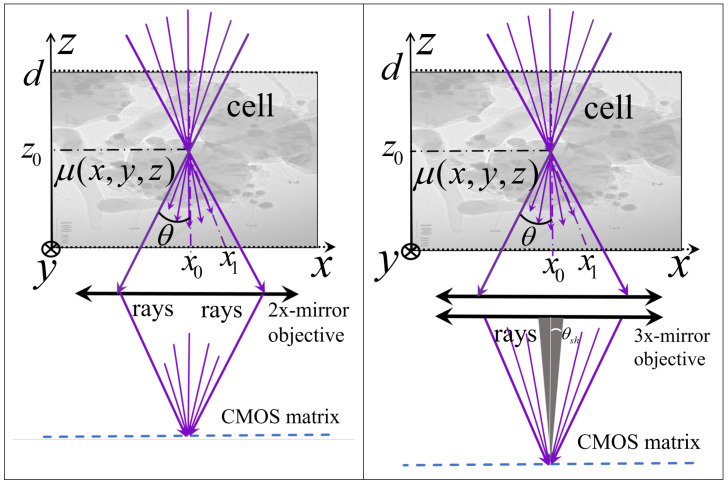
Beam diagrams of ×46 (**left**) and ×345 (**right**) X-ray microscopes passing through samples in the lens focal plane, forming magnified images of all focal plane points on the detector. *θ* is the angle of the light cone; *θ_sh_* if the angle of the shadow.

Figure 2 presents simplified beam patterns in these microscopes.

Signal intensity at each matrix point is determined by all rays from the cone passing through the corresponding focal point (*x*_0_, *y*_0_, *z*_0_) and exiting at points (*x*_1_, *y*_1_, 0) in the measurement chamber (Figure 1). A mirror lens constructs an image of the sample slice in the lens focal plane. To obtain 3D signal distributions, samples are moved along the *z*-axis (lens optical axis), and a series of 2D *xy* signal distributions (*z*-stack) are recorded on the matrix. The pixel size is obtained by the transverse diffraction spreading of the focused radiation. Table 1 presents the key experimental parameters implemented in cell reconstructions using the ×46 and ×345 EUV microscopes.

### 2.2. Tomography Methods

For the ×46 EUV microscope Figure 2 (left), with 140 nm object plane pixel size, we formulated and solved the inverse tomography problem based on inverse Radon transformation in the weak absorption geometric-optical approximation [13,14]. For the obtained 3D convolution-type inverse tomography problem integral equation, an algorithm that reconstructs the 3D absorption coefficient distribution was developed. Numerical simulation and algorithm application to plant-cell samples demonstrated single-pixel point object (140 nm) resolution. However, since absorption in most cells did not satisfy the low absorption condition, the quantitative analysis in [14] inevitably contained corresponding error.

This article presents a theory generalization [13,14] for the geometric-optical approximation of small inhomogeneities in absorbing media. This generalization significantly expands the applicability of tomographic diagnostics, enabling more accurate 3D reconstruction of the absorption coefficient distribution in plant cells.

For the new ×345 EUV microscope with 30 nm Rayleigh resolution limit, measurements were taken with 19 nm linear pixel size in *x* and *y* directions and 50 nm in the *z*-direction (determined by the chosen scanning step of piezoceramic actuator (manufactor Physik Instrumente, Karlsruhe, Germany). To reduce high random error levels and adjust pixel sizes in horizontal and vertical coordinates (important for fast Fourier transform application), sliding averaging of the 680 × 680 × 160 distribution to a 256 × 256 × 160 grid with 50 nm pixel size was performed for all coordinates. During tomography algorithm development, we considered that the two-mirror lens introduces aberrations from slight mirror shape imperfections (~1 nm rms level). Additionally, the cone-shaped light beam entering the lens from each focal plane point is partially obscured by the lens convex mirror (Figure 2). To account for these effects, we applied the kernel determination method based on experiments with known test objects, similar to our previous approach [15], assuming that signal formation in weakly absorbing objects is still determined by the 3D convolution equation. Results confirmed this approach’s effectiveness.

### 2.3. Biological Samples

Plant cells (Lily of the valley stem, *Convallaria*). This is a standard dried-lily-of-the-valley stem preparation manufactured by LIEDER (Karlsruhe, Germany) for optical confocal microscopy. For observation in the EUV microscope, the specimen film was separated from the coverslip by soaking in acetone and then transferred onto the surface of a silicon nitride membrane.

Neurites of mouse cerebellar granule cells were isolated from 7–8-day old Wistar rat pups, dissociated by pipetting in culture medium, and plated onto poly-L-lysine-coated silicon nitride membrane surfaces (Norcada, Edmonton, AB, Canada). After three days of cultivation in a CO_2_ incubator, cells were fixed with a mixture of formaldehyde and glutaraldehyde (4% + 1%) in PBS (pH 7.4), dehydrated through a graded ethanol series and acetone, and dried at the critical point of CO_2_ similarly to preparations for scanning electron microscopy.

## 3. Results

### 3.1. EUV Microscope with ×46 Magnification

#### 3.1.1. Theory

This work achieves significant generalization of the geometric-optical tomography theory developed in [13,14], extending it to weak inhomogeneities in absorbing media. We have also developed an appropriate tomography algorithm based on this theory. As in [14], the theory starting point is the Radon transform for the measurement geometry under consideration.

The analysis is based on the radiation transport equation in an absorbing medium, where the intensity at the absorbing layer exit is determined by optical depth $\tau =\int_{L}\mu (x,y,z)dl$, which is the integral of the absorption coefficient along the beam path on a straight line passing through points *x*_0_, *y*_0_, *z*_0_ and *x*_1_, *y*_1_, *z*_1_ = 0 between planes *z* = 0 and *z* = *d* (see in Figure 2):

(1) $$J(x_{1},y_{1},z_{1}=0,x_{0},y_{0},z_{0})=J_{0}e^{-\int_{L}\mu (x,y,z)dl}.$$

Using the integral parametric representation in (1) and integrating over all rays within the cone exiting through the *z* = 0 plane, we can obtain the direct problem solution, which is the signal intensity ratio with and without the analyzed object $I(x_{0},y_{0},z_{0})/I_{0}[\mu =0]$ at the corresponding point of the CMOS matrix (Radon transformation in the measurement geometry under consideration) [13]:

(2) $$\begin{array}{l} I(x_{0},y_{0},z_{0})/I_{0}[\mu =0]=\int_{x_{0}-z_{0}\tan\theta }^{x_{0}+z_{0}\tan g\theta }dx_{1}\int_{-\sqrt{{\left(z_{0}\tan\theta \right)}^{2}-{\left(x_{0}-x_{1}\right)}^{2}}}^{\sqrt{{\left(z_{0}\tan\theta \right)}^{2}-{\left(x_{0}-x_{1}\right)}^{2}}}dy_{1}\\ \;\;\times e^{-\sqrt{{\left(x_{0}-x_{1}\right)}^{2}+{\left(y_{0}-y_{1}\right)}^{2}+{z_{0}}^{2}}/z_{0}\int_{0}^{d}\mu [x_{1}+(x_{0}-x_{1})z/z_{0},\;y_{1}+(y_{0}-y_{1})z/z_{0},\;z]dz}/\;\int_{x_{0}-z_{0}\tan\theta }^{x_{0}+z_{0}\tan\theta }dx_{1}2\sqrt{{\left(z_{0}\tan\theta \right)}^{2}-{\left(x_{0}-x_{1}\right)}^{2}}. \end{array}$$

The most well known methods of medical tomography, CT (X-ray computed tomography) and MRI (magnetic resonance imaging), also lead to similar but different Radon transformations because, unlike axial tomography considered here, these methods use circular scanning. These methods are based on solving corresponding inverse problems in cylindrical coordinates, which creates specific challenges when transforming to images in Cartesian coordinates. Their theory was first developed by Radon [16] and later applied in computed X-ray tomography by A.N. Tikhonov [17], in line with his theory of incorrect inverse problems.

Unlike CT and MRI, the initial equation in this problem is nonlinear, creating serious theoretical and computational challenges. In the solution method [13,14], the problem was solved in the small optical absorption thickness approximation *τ* << 1, enabling exponential decomposition and obtaining a linear 3D convolution-type integral equation for the desired distribution *μ*(*x*, *y*, *z*). In this approximation, for relative intensity decrease, we obtain the expression [13,14]:

(3) $$\begin{array}{l} \delta I(x_{0},y_{0},z_{0})=[I_{0}-I]/I_{0}=\;\int_{x_{0}-z_{0}tg\theta }^{x_{0}+z_{0}tg\theta }dx_{1}\int_{-\sqrt{{\left(z_{0}tg\theta \right)}^{2}-{\left(x_{0}-x_{1}\right)}^{2}}}^{\sqrt{{\left(z_{0}tg\theta \right)}^{2}-{\left(x_{0}-x_{1}\right)}^{2}}}dy_{1}\sqrt{{\left(x_{0}-x_{1}\right)}^{2}+{\left(y_{0}-y_{1}\right)}^{2}+{z_{0}}^{2}}/z_{0}\;\;\;\;\;\;\;\;\;\;\;\;\;\;\;\;\;\;\;\;\;\\ \;\;\;\;\;\;\;\;\;\;\;\;\;\;\;\;\times \int_{0}^{d}\mu [x_{1}+(x_{0}-x_{1})z/z_{0},\;y_{1}+(y_{0}-y_{1})z/z_{0},\;z]/\int_{x_{0}-z_{0}tg\theta }^{x_{0}+z_{0}tg\theta }dx_{1}2\sqrt{{\left(z_{0}tg\theta \right)}^{2}-{\left(x_{0}-x_{1}\right)}^{2}}dz. \end{array}$$

After transformations and integration over variables *x*_1_, *y*_1_, a 3D convolution tomography equation is obtained [13,14]:

(4) $$\delta I(x_{0},y_{0},z_{0})=[I_{0}-I]/I_{0}=\int_{0}^{d}\iint \mu (x,y,z)K(x_{0}-x,y_{0}-y,z_{0}-z)dxdydz,$$

(5) $$K(x_{0}-x,y_{0}-y,z_{0}-z)=\left\{\begin{array}{l} =\frac{2}{\pi {\left(tg\theta \right)}^{2}{\left(z_{0}-z\right)}^{2}}\sqrt{{\left(\frac{x_{0}-x}{z_{0}-z}\right)}^{2}+{\left(\frac{y_{0}-y}{z_{0}-z}\right)}^{2}+1}\\ \mathrm{at}\;\left|\frac{x_{0}-x}{z_{0}-z}\right|<tg\theta ,\;\left|\frac{y_{0}-y}{z_{0}-z}\right|<\sqrt{{\left(tg\theta \right)}^{2}-{\left(\frac{x_{0}-x}{z_{0}-z}\right)}^{2}};\\ =0\;\mathrm{at}\;\left|\frac{\left(x_{0}-x\right)}{z_{0}-z}\right|>tg\theta ,\;\left|\frac{y_{0}-y}{z_{0}-z}\right|>\sqrt{{\left(tg\theta \right)}^{2}-{\left(\frac{x_{0}-x}{z_{0}-z}\right)}^{2}} \end{array}\right\},$$

where the conditions for finding radiation within the cone are also included in the kernel of the equation *K*. Three-dimensional Fourier transform of (4) yields a simple *k*-space spectrum equation:

(6) $$\delta I(k_{x},k_{y},k_{z})=8\pi^{3}\mu (k_{x},k_{y},k_{z})K(k_{x},k_{y},k_{z}),$$

and the Radon inverse transformation formula for this tomography method:

(7) $$\mu (x,y,z)=\;\frac{1}{8\pi^{3}}∭\delta I(k_{x},k_{y},k_{z})/K(k_{x},k_{y},k_{z})e^{ik_{x}x+ik_{y}y+ik_{z}}dk_{x}dk_{y}dk_{z},$$

where Fourier transforms use the same notation as transformed parameters, differing only in arguments.

This enabled algorithm development is based on inverse Fourier transform [13]. During algorithm development, we had to address the problem of kernel divergence in Equation *K* (5) at the focal point. In [13], the value of *K* at the focal point was chosen by extrapolation from neighboring pixels, and the algorithm was tested in numerical simulations on a 20 × 20 × 20 grid. Due to small dimensionality, test inhomogeneities exhibited noticeable blurring. Significant discretization errors led to the amplification of simulated random errors, i.e., the manifestation of ill-posedness, which required the application of Tikhonov regularization.

In our subsequent paper [14], the algorithm was refined for application to plant-cell analysis using data from measurements with the ×46 microscope. Measurements were performed with 256 × 256 × 219 discretization at a pixel size of 140 nm, chosen based on the scale of diffraction-related beam blurring. It turned out that the choice of kernel value at the focal pixel made in [13] was impractical. Varying this parameter allowed us to determine its optimal value by maximizing the observed resolution of the smallest details in reconstructions of the studied objects. As a result, 140 nm resolution of observed organelles was achieved in reconstructions of relatively transparent cell regions.

Numerical simulations showed that the algorithm does not exhibit properties of an ill-posed problem—it does not amplify added random errors and accurately reproduces test objects without added errors. Apparently, this property is due to the presence of a weak singularity in the kernel of the equation being solved—such problems, like the inverse Abel transform, which has an exact solution, can possess this feature. Therefore, regularization was not applied in the cell analysis presented in [14]. This specificity of axial tomography is an important advantage compared to standard CT.

Unfortunately, the conditions of low absorption were not satisfied in most of the volume of the studied cells, which limited the accuracy of the determined absorption coefficient of the observed inhomogeneities.

This paper obtains significant theory generalization for weak inhomogeneities in absorbing media and develops an appropriate tomography algorithm. The tomography-integral equation is derived from Formula (1), which describes radiation intensity in the geometric-optical approximation, assuming absorption coefficient inhomogeneity is small compared to the constant absorption component in the analyzed region $\mu =\mu_{0}+\mu_{1},\;\mu_{1}<<1$:

(8) $$\begin{array}{l} I(x_{0},y_{0},z_{0})/I_{0}[\mu =0]=\int_{x_{0}-z_{0}\tan\theta }^{x_{0}+z_{0}\tan\theta }\int_{y_{0}-\sqrt{{\left(z_{0}\tan\theta \right)}^{2}-{\left(x_{0}-x_{1}\right)}^{2}}}^{y_{0}+\sqrt{{\left(z_{0}\tan\theta \right)}^{2}-{\left(x_{0}-x_{1}\right)}^{2}}}e^{-\mu_{0}\sqrt{{\left(x_{0}-x_{1}\right)}^{2}+{\left(y_{0}-y_{1}\right)}^{2}+{z_{0}}^{2}}d/z_{0}}dx_{1}dy_{1}\\ /\int_{x_{0}-z_{0}\tan\theta }^{x_{0}+z_{0}\tan\theta }2\sqrt{{\left(z_{0}\tan\theta \right)}^{2}-{\left(x_{0}-x_{1}\right)}^{2}}dx_{1}\\ \;\;-\int_{x_{0}-z_{0}\tan\theta }^{x_{0}+z_{0}\tan\theta }dx_{1}\int_{y_{0}-\sqrt{{\left(z_{0}\tan\theta \right)}^{2}-{\left(x_{0}-x_{1}\right)}^{2}}}^{y_{0}+\sqrt{{\left(z_{0}\tan\theta \right)}^{2}-{\left(x_{0}-x_{1}\right)}^{2}}}dy_{1}e^{-\mu_{0}\sqrt{{\left(x_{0}-x_{1}\right)}^{2}+{\left(y_{0}-y_{1}\right)}^{2}+{z_{0}}^{2}}d/z_{0}}\sqrt{{\left(x_{0}-x_{1}\right)}^{2}+{\left(y_{0}-y_{1}\right)}^{2}+{z_{0}}^{2}}/z_{0}\\ \int_{0}^{d}\mu_{1}[x_{1}+(x_{0}-x_{1})z/z_{0},\;\;y_{1}+(y_{0}-y_{1})z/z_{0},\;z]dz/\int_{x_{0}-z_{0}tg\theta }^{x_{0}+z_{0}tg\theta }dx_{1}2\sqrt{{\left(z_{0}\tan\theta \right)}^{2}-{\left(x_{0}-x_{1}\right)}^{2}}\;. \end{array}$$

By performing transformations and substitutions of variables, similar to those described in [13], and including the limits of integration in the kernel of the equation, we obtain a 3D convolution-type integral tomography equation with a weak singularity kernel. This equation can be expressed explicitly as:

(9) $$\delta I(x_{0},y_{0},z_{0})=\frac{I(\mu_{0})}{I_{0}}-\frac{I}{I_{0}}(x_{0},y_{0},z_{0})=\int_{0}^{d}\iint \mu_{1}(x,y,z)K(x_{0}-x,y_{0}-y,z_{0}-z)dxdydz,$$

(10) $$K(x_{0}-x,y_{0}-y,z_{0}-z)=\left\{\begin{array}{l} =\frac{\sqrt{{\left(\frac{x_{0}-x}{z_{0}-z}\right)}^{2}+{\left(\frac{y_{0}-y}{z_{0}-z}\right)}^{2}+1}}{\pi {\left(t\tan\theta \right)}^{2}{\left(z-z_{0}\right)}^{2}}e^{-\mu_{0}d\sqrt{{\left(\frac{x_{0}-x}{z_{0}-z}\right)}^{2}+{\left(\frac{y_{0}-y}{z_{0}-z}\right)}^{2}+1}\;},\\ \left|\frac{x_{0}-x}{z_{0}-z}\right|<\tan\theta ,\;\left|\frac{y_{0}-y}{z_{0}-z}\right|<\sqrt{{\left(\tan\theta \right)}^{2}-{\left(\frac{x_{0}-x}{z_{0}-z}\right)}^{2}}\\ =0,\;\;\;\;\left|\frac{x_{0}-x}{z_{0}-z}\right|>\tan\theta ,\;\left|\frac{y_{0}-y}{z_{0}-z}\right|>\sqrt{{\left(\tan\theta \right)}^{2}-{\left(\frac{x_{0}-x}{z_{0}-z}\right)}^{2}} \end{array}\right\}$$

where $\frac{I}{I_{0}}(x_{0},y_{0},z_{0})$ is the measured signal intensity distribution ratio to signal without object depending on focus position, and $\frac{I(\mu_{0})}{I_{0}}$ is a theoretical correction accounting for the medium constant absorption component, explicitly represented by the first term on Equation (8), right-hand side:

(11) $$\begin{array}{l} \;\;\frac{I(\mu_{0})}{I_{0}}=\frac{1}{\tan^{2}\theta }\int_{0}^{\tan^{2}\theta }e^{-d\mu_{0}\sqrt{x+1}}dx=\\ =2\frac{e^{-d\mu_{0}}-d\mu_{0}\sqrt{\tan^{2}\theta +1\;}e^{-d\mu_{0}\sqrt{\tan^{2}\theta +1}}-e^{-d\mu_{0}\sqrt{\tan^{2}\theta +1}}+d\mu_{0}e^{-d\mu_{0}}}{\tan^{2}\theta {\left(d\mu_{0}\right)}^{2}}, \end{array}$$

where *d* is the vertical size of the analysis area and θ is the angle between the ray cone generatrix and the vertical axis. This simple formula has independent interest, as it determines the signal from a layer with a constant absorption coefficient, enabling inverse problem solution: determining the homogeneous layer absorption coefficient from the measured signal. This has obvious practical significance and can be used for calibration in our proposed tomography method.

Comparing kernel functions in (5) and (10), in (10), a multiplier accounting for the constant absorption component has appeared. At $\mu_{0}$ = 0, these formulas coincide.

Three-dimensional Fourier transform of Equation (9) leads to a *k*-space spectrum equation similar to (6):

(12) $$\delta I(k_{x},k_{y},k_{z})=8\pi^{3}\mu_{1}(k_{x},k_{y},k_{z})K(k_{x},k_{y},k_{z})$$

and its Cartesian coordinate solution is expressed as:

(13) $$\mu (x,y,z)=\mu_{0}+\mu_{1}(x,y,z)=\mu_{0}\;+\frac{1}{8\pi^{3}}∭\delta I(k_{x},k_{y},k_{z})/K(k_{x},k_{y},k_{z})e^{ik_{x}x+ik_{y}y+ik_{z}}dk_{x}dk_{y}dk_{z}.$$

Generally, solving inverse problems such as convolution using input data containing errors can be ill-posed. Random errors can have broader spatial spectra than the kernel, leading to uncontrolled small-scale feature amplification in solutions. To address this, Tikhonov regularization [17] can be applied when necessary.

In the proposed method (9)–(13), in which the kernel *K* in (10) has the same divergence as the above-considered kernel in (5), more accurate scaling is achieved by using the ratio (11) between the signal from vertically homogeneous layers of the objects under study and their absorption coefficient.

**Figure 3:**
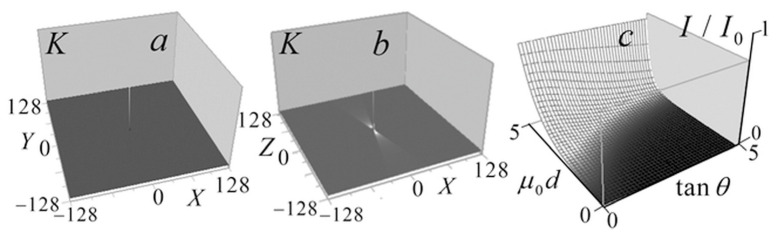
(**a**) Horizontal section of the kernel function (10) in Equation (9); (**b**) kernel *K* in a vertical section; (**c**) the distribution of the correction term (11).

Figure 3 shows the kernel *K* (10) of the tomography Equation (9), and the distribution of the correction term (11) in the left-hand side of this equation, depending on the arguments $\left(\mu_{0}d,\tan\theta \right)$.

Unlike the kernel in the low-absorption approximation [13,14], in this case, this kernel distribution is not universal but depends on the analyzed object absorption coefficient constant component $\mu_{0}$. The kernel peak (Figure 3a) manifests as a discrete *δ*-function, forming a pedestal in its spatial spectrum, ensuring method resolution down to the smallest scales determined by signal blurring in focus. The correction term distribution in Figure 3c shows a decrease with increasing absorption and light-cone angle θ. The one-to–one correspondence between $I/I_{0}$ and the argument $\mu_{0}d$ for any angle θ enables considering this distribution as an inverse problem solution for determining $\mu_{0}\;(I/I_{0})$, i.e., as a method for determining the homogeneous layer absorption coefficient of $\mu_{0}$ from the measured signal attenuation $I/I_{0}$.

This result is important not only as a method for determining the absorption coefficient of homogeneous layers, but also as a calibration method for 3D reconstructions in axial tomography. If a quasi-homogeneous layer ($I/I_{0}\approx const$) can be identified in the analyzed object, we determine its absorption coefficient $\mu_{l}\;(I/I_{0})$ and choose the focal kernel value in (10) such that the absorption of this layer in the reconstruction corresponds to $\mu_{l}$. This value, together with the value $\mu_{0}\;(I/I_{0})$ of the constant component of the absorption distribution, determines the scale for calibrating the reconstruction (13). If necessary, an artificial layer with a known absorption coefficient can be placed together with the object.

#### 3.1.2. Numerical Simulation

When developing algorithms based on solving inverse problems described by integral equations, solution accuracy is not directly proportional to the error level and depends on the specifics of the structure of a particular analyzed object. Additionally, artifacts may arise during reconstruction. Therefore, numerical simulation plays a crucial role as a necessary research stage.

**Figure 4:**
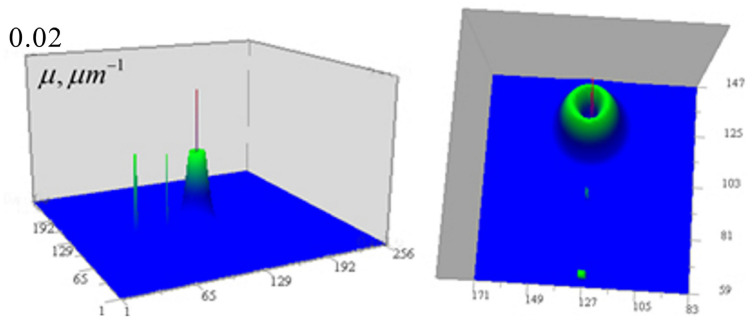
Inhomogeneities of the absorption coefficient used in numerical modeling.

For the tomography algorithms (6)–(10), numerical simulations were performed for test objects with given geometric structure (point-like, homogeneous in absorption coefficient, and heterogeneous modeled by Gaussian distributions). The study was conducted using a closed loop: (a) for each focus position, the 3D received signal distribution is calculated; (b) a random Gaussian “measurement error” with zero mean and a specified standard deviation is added to the signal; (c) the inverse problem (9–10) is solved; (d) the resulting solution is compared with the initial distribution. Figure 4 shows the results of numerical simulation of algorithms (9) and (10) for test inhomogeneities in a medium with a constant component $\mu_{0}$ = 0.08 µm^−1^ (the optical thickness of such a medium is $\mu_{0}d=1.024$, and $I/I_{0}(\mu d)=0.353$). Inhomogeneities of the absorption coefficient were modeled: distributed inhomogeneity based on a Gaussian distribution with a Gaussian internal cavity

$$\mu_{1}(x,y,z)=0.02\exp[-\frac{{\left(x-x_{c}\right)}^{2}+{\left(y-y_{c}\right)}^{2}+{\left(z-z_{c}\right)}^{2}\;}{\sigma_{1}^{2}}]-\exp[-\frac{{\left(x-x_{c}\right)}^{2}+{\left(y-y_{c}\right)}^{2}+{\left(z-z_{c}\right)}^{2}\;}{\sigma_{2}^{2}}]\;{µm}^{-1},$$

where *x*_c_ = *y*_c_ = *z*_c_ = 128 px, *σ*_1_ = 4 px, *σ*_2_ = 2 px (1 px = 50 nm); cube = 0.01 µm^−1^ with dimensions 3 × 3 × 3 px at *x*_c_ = 64 px, and two 1-pixel inhomogeneities: $\mu_{1}(96,128,128)$ = 0.02 µm^−1^, $\mu_{1}(128,128,128)$ = 0.01 µm^−1^. These inhomogeneities have the same appearance in the *xy* and *xz* sections and are shown in Figure 4 from two angles.

**Figure 5:**
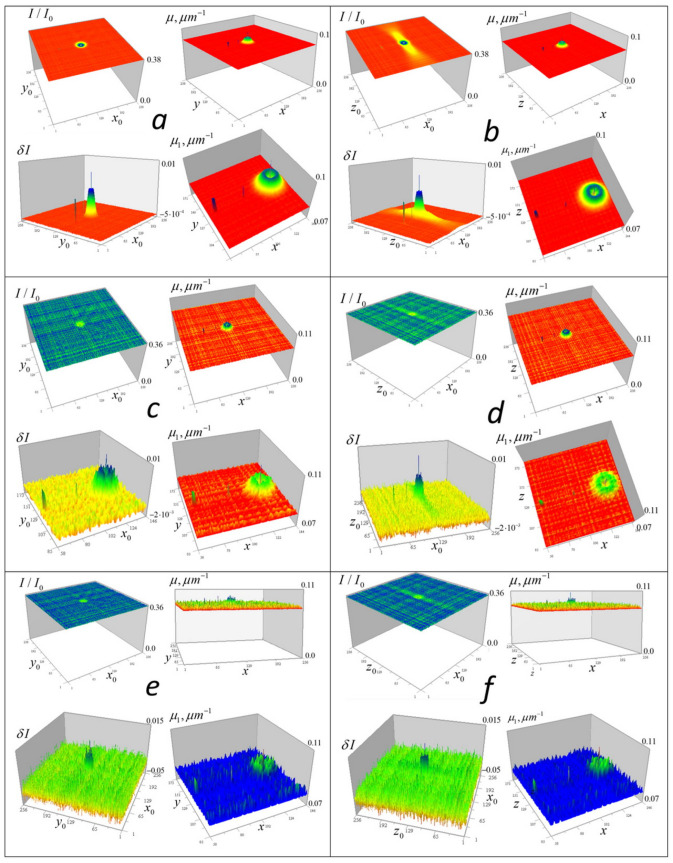
Numerical modeling of tomographic reconstruction. The left column shows the results in horizontal *xy* section $z=128\;(z_{0}=128)$; the right column shows the results in vertical (along the optical axis of the lens) cross-section y=128 $\left(y_{0}=128\right)$. Reconstructions (**a**,**b**) were obtained at a simulated random error level σI = 1% with the maximum of δI for the heterogeneity based on Gaussian functions. Reconstructions (**c**,**d**) were obtained with an error level σI = 10% and reconstructions (**e**,**f**) were obtained with a 30% error level.

Figure 5 shows numerical simulation results obtained without regularization at simulated random error levels of σ = 1, 10, and 30% of the maximum value of the large Gaussian test inhomogeneity.

Figure 5 shows the full-scale (256 × 256) received signal distributions $I/I_{0}$ with added random error and corresponding enlarged (128 × 128) distributions δI of the left side of the tomography Equation (9), which displays the contribution of the test objects to the signal. It also shows the tomographic distributions of the absorption coefficient μ of the test objects obtained from these data (in full-scale) and the contribution of the test objects to this distribution (128 × 128, above the level of the constant component $\mu_{0}$). The color scale was chosen for optimal distinguishability of inhomogeneities.

In Figure 5, it can be seen that test objects make small contributions to the distribution of signal attenuation $I/I_{0}$ compared to the constant component of the absorption coefficient *μ*_0_. However, with small (1%) error in δI (Figure 5a,b), they are reproduced almost accurately, including a small cube with sharp edges, an internal cavity in a Gaussian function solid object, and two 1-pixel inhomogeneities—one inside this cavity and another outside the Gaussian object. It is important to note that despite the significant signal blurring in the vertical plane (right column of Figure 5), corresponding tomographic reconstruction results do not differ significantly from horizontal plane reconstructions over the wide data error range shown in Figure 5.

In reconstructions obtained without regularization, errors appear almost proportional to the simulated random error levels, which confirms the correctness of the inverse problem formulation. As seen in Figure 5e,f, even with a 30% error rate, the small cube and 1-pixel objects are reconstructed well—the method implements such resolution for inhomogeneities whose signal exceeds noise level. However, numerical simulation results do not yet permit conclusions about the same effectiveness in real diagnostics.

#### 3.1.3. Cell Tomography with ×46 EUV Microscope

Experimental studies of this tomography method using ×46 EUV microscope were conducted on dried plant cells (lily of the valley stem, *Convallaria*)—objects with a complex, multiscale internal structure, sampled at 256 × 256 × 160 with a resolution of 140 nm. These cell reconstructions by the weak absorption approximation algorithm were presented in [14], enabling comparison with new algorithm results for inhomogeneities in the absorbing medium (9–13).

**Figure 6:**
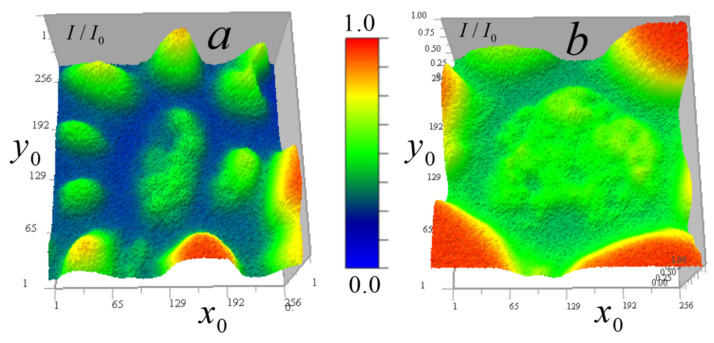
Distribution of the measured signal $\frac{I}{I_{0}}(x_{0},y_{0})$ in the horizontal plane 256 × 256 pixels (36 × 36 μm^2^) for two *Convallaria* cells: (**a**) cell *A* at level $z_{0}$ = 108; (**b**) distribution for cell *B* at level $z_{0}$ = 174.

Figure 6 shows the measured signal distribution from two cells in the horizontal plane.

**Figure 7:**
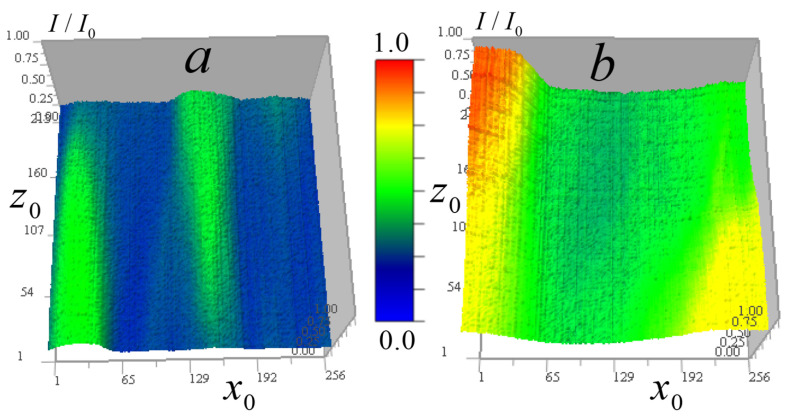
Measured signal $\frac{I}{I_{0}}(x_{0},z_{0})$ distribution in the vertical plane 256 × 213 pixels (36 × 30 μm^2^) for two cells: (**a**) cell *A* at $y_{0}$ = 170; (**b**) distribution for cell *B* at $y_{0}$ = 53.

Figure 7 shows these cells in the vertical plane.

Figure 6 and Figure 7 demonstrate that cell *B* has generally higher transparency and more uniform distribution than cell *A*. In the cell *A* central region, the lowest vertical absorption can be observed at the point (97, 170, 108). This enables determining a constant absorption coefficient component for this region by relative signal attenuation at this point $\mu_{0}(I/I_{0})$ = 0.0245 μm^−1^. Therefore, at this point $\mu_{1}(97,170,108)=\mu -\mu_{0}$ = 0.

The cells in Figure 7 have vertical layers with nearly uniform vertical attenuation $I/I_{0}$, and therefore, the corresponding distribution of the absorption coefficient is close to a constant value. This enables determining the absorption coefficient of the layer, selecting a second calibration point for the reconstruction result (13) and adjusting the kernel focus based on the known absorption in this layer. In cell *A*, a point (97, 170, 108) was selected for calibration in an almost vertically homogeneous cell area with relatively high absorption, for which $\mu =\mu_{0}(I/I_{0})$ = 0.0449 µm^−1^. The distribution below and above the point is nearly uniform—variations are insignificant (the standard deviation on the vertical line passing through this point is less than 2%). For cell *B*, the value of the constant component determined by the minimum absorption $I/I_{0}$(205,107,174) = 0.624 in a homogeneous layer was $\mu_{0}(I/I_{0})$ = 0.0157 µm^−1^. A second calibration point (87, 53, 174) was selected from a layer with relatively high absorption in this region $\mu (I/I_{0})$ = 0.0313 µm^−1^.

**Figure 8:**
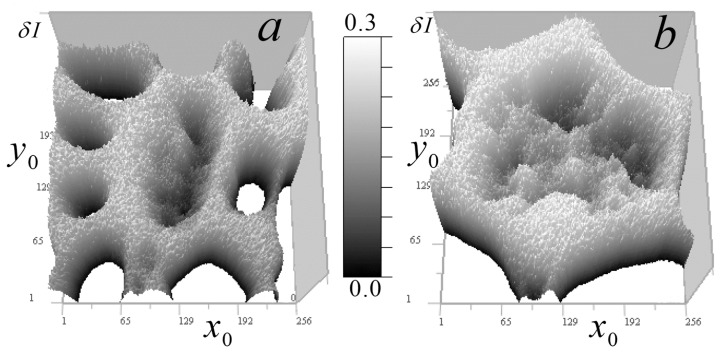
Distribution $\delta I(x_{0},y_{0})$ on the 256 × 156 pixels (36 × 36 µm^2^) horizontal plane for two *Convallaria* cells: (**a**) cell *A* at level $z_{0}$ = 108, (**b**) cell *B* at $z_{0}$ = 174.

In Figure 8, one can see the signal regions highlighted above the constant component, which allows one to obtain more accurate quantitative data on small inhomogeneities in these regions.

**Figure 9:**
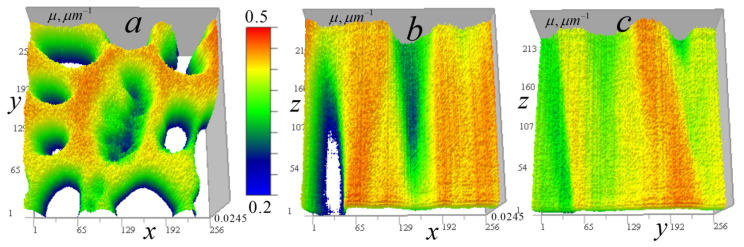
Reconstruction of the absorption coefficient *μ* in cell *A*: (**a**) section in region 256 × 256 pixels (36 × 36 μm^2^) in the horizontal plane *xy* (z = 108); (**b**,**c**)—sections 256 × 260 pixels (36 × 30 μm^2^) in the vertical planes *xz* (*y* = 170) and *yz* (*x* = 97), respectively.

Figure 9 shows the results of the reconstruction of the absorption coefficient distribution in both horizontal and vertical tomographic sections for cell *A*.

**Figure 10:**
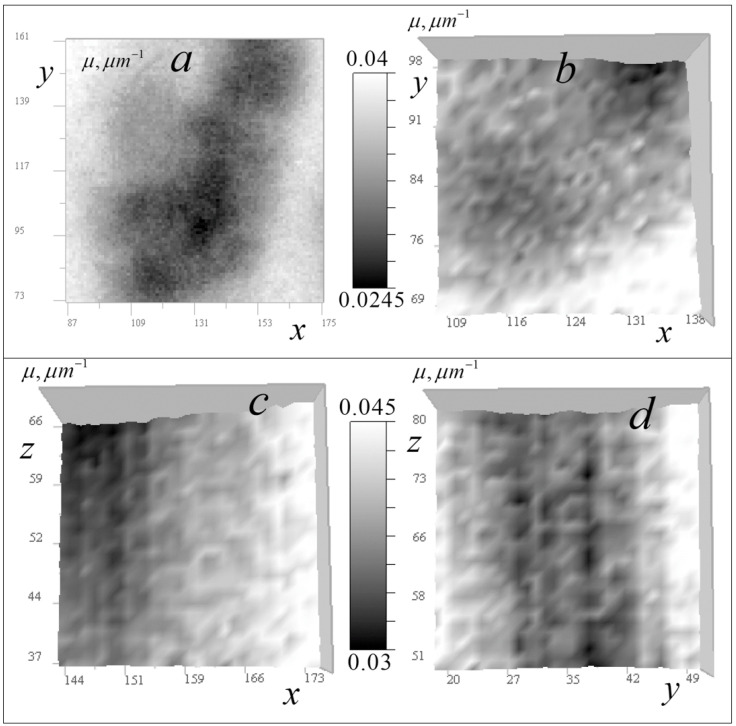
Reconstruction of the absorption coefficient in cell *A*: (**a**,**b**) tomographic sections (12.5 × 12.5 μm^2^) in the horizontal *xy* plane at *z* = 108; (**c**,**d**) sections (4.2 × 4.2 μm^2^) in the vertical *xz* planes at *y* = 170 and (*yz*) at *x* = 97.

In Figure 10, the results of the tomography are shown at a reduced scale, demonstrating, in comparison with the results of [14], a greater depth of contrast and improved detail of the smallest distinguishable organelles visible in areas of relative transparency. Numerous small ring-shaped absorption inhomogeneities with dimensions 0.003–0.007 micrometers are visible. Presumably, they are shells of spheroidal bodies with a thickness of 0.15–0.3 μm, more transparent contents (by 0.0005–0.003 μm^−1^), and transverse dimensions of 0.3–0.7 μm. Rings shown in the horizontal section in Figure 10a,b are, on average, smaller than rings shown in vertical sections in Figure 10c,d.

**Figure 11:**
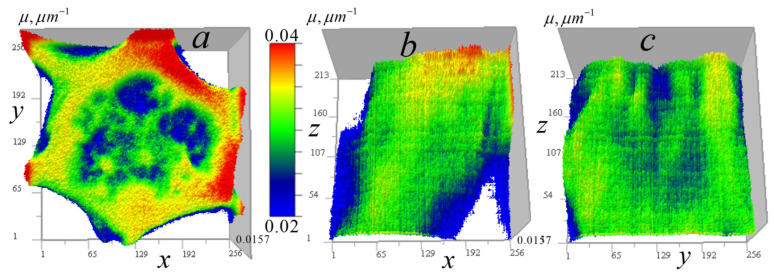
Reconstruction of the absorption coefficient in cell *B*: (**a**) tomographic section 256 × 256 pixels (36 × 36 μm^2^) in the horizontal plane *xy* (*z* = 174); (**b**,**c**) sections 256 × 213 pixels (36 × 30 μm^2^) in the vertical planes *xz* at *y* = 53 and *yz* at *x* = 87.

Figure 11 shows the results of the tomographic reconstruction of the absorption coefficient distribution in cell B, both horizontally and vertically (in vertical sections passing through calibration points with relatively high absorption).

**Figure 12:**
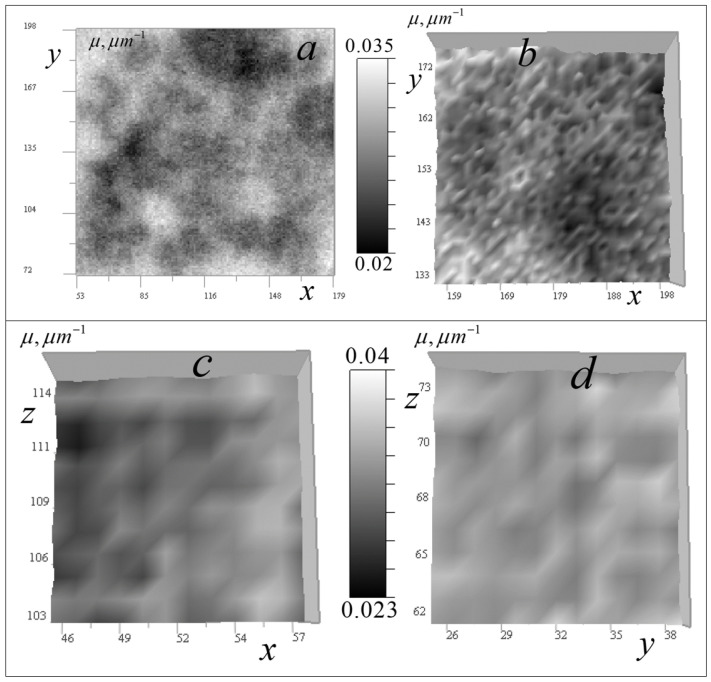
Reconstruction of the absorption coefficient in cell *B*: (**a**,**b**) tomographic sections ((**a**)*:* 17.8 × 17.8 μm^2^, (**b**): 5.6 × 5.6 μm^2^) in the horizontal *xy* plane (*z* = 174); (**c**,**d**) sections of 1.7 × 1.7 μm^2^ in the vertical *xz* (*y* = 53) and *yz* (*x* = 87) planes.

Figure 12 shows cell *B* tomography results at a reduced scale.

In Figure 12, tomography results also show an improvement in the detail of the smallest organelles compared to reconstructions in [14]. In the horizontal section of a relative transparency area in cell B (Figure 12a), ordered star-shaped formations and various-sized ring structures are visible. Figure 12b shows a small area with accumulation of the smallest annular bodies, with linear dimensions ranging from 0.3 to 0.7 μm with a shell thickness ranging from 0.15 to 0.3 μm, which were not found in the reconstructions [14]. The absorption coefficient variations in these bodies are comparable to those seen in the reconstruction shown in Figure 10b.

Figure 12c,d show two vertical sections of the largest ring formations, with transverse dimensions of approximately 0.7 μm, shell thicknesses up to 0.3 μm, and cavity dimensions ranging from 0.3 to 0.45 μm. In the cell in Figure 12c, the ring organelles’ absorption range is 0.0279–0.0291 μm^−1^ and inner cavity is 0.0256–0.0267 μm^−1^, i.e., the shell’s contribution to absorption is 0.0023–0.0035 μm^−1^. There is also an internal inclusion with absorption of 0.0273 μm*^−^*^1^. In the cell shown in Figure 12d, absorption is higher: ring organelles’ ranges are 0.0312–0.0326 μm^−1^, cavity is 0.0300–0.0308 μm^–1^, and the contribution from the shell is less than 0.0004–0.0026 μm^−1^. There is a small inclusion with absorption of 0.0306 μm^−1^ that is not easily visible.

**Figure 13:**
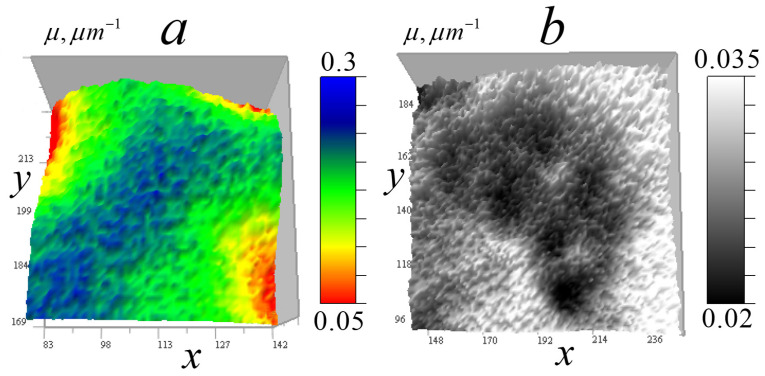
Cell absorption coefficient reconstruction: (**a**) tomographic cross-section 60 × 60 pixels (8.4 × 8.4 μm^2^) of cell *A* in the *xy* plane (*z* = 108) in the Rainbow Inverted color scale; (**b**) cross-section 89 × 89 pixels (12.5 × 12.5 μm^2^) of cell *B* in the *xy* plane (*z* = 60) in grayscale.

Figure 13 shows two reconstructions for cells *A* and *B*, respectively. Such structures were not resolved in [14] using the algorithm in the weak heterogeneity approximation.

Using the selected color scale, ring-shaped structures (some with absorbing nuclei) against an increased absorption background (~0.2 μm^−1^) were revealed in cell *A*. The absorption coefficient in the nuclei exceeded that of the more transparent environment by 0.001–0.003 μm^−1^. By choosing a contrast in the tomogram of cell *B*, thin details of large-scale star-shaped organelles can be identified. Such details were indistinguishable in reconstructions obtained in the small absorption approximation [14].

Thus, the tomograms in Figure 9, Figure 10, Figure 11, Figure 12 and Figure 13 reveal the complex internal structure of the absorption coefficient distribution within cells—quantitative information inaccessible to optical diagnostic techniques, certainly of interest to biologists. The cellular structure in the images contains localized elements of varying scales. The ring formations, which are presumably parts of spherical bodies in the cell, are of particular interest. The resolution of the presented method enables detailed analysis of these small intracellular structures. Results demonstrate that the tomography algorithm provides a resolution of 1 pixel (140 nm) with the ×46 EUV microscope.

### 3.2. EUV Microscope with ×345 Magnification

#### 3.2.1. Novel Empirically Derived Tomography Method

In 2025, we developed a new ×345 X-ray microscope with a magnification 7.5 times greater than that of the ×46 microscope discussed above, which opens opportunities for more detailed tomography. However, due to aforementioned problems with the direct application of geometric-optical algorithms for this microscope, we have developed an alternative method that takes into account the features of the new objective. In this approach, assuming that for weakly absorbing objects, the signal formation can still be described by a 3D convolution-type equation, we used a method that determined its kernel from an experiment with test objects having a known shape, position, and absorption coefficient. We have previously used a similar approach in microwave subsurface tomography [15].

Gold balls with 100 and 300 nm diameters and an absorption coefficient of 0.19 μm^−1^ were selected as such test objects. These objects were placed on a transparent surface. Sphere shape and position of the balls were determined using electron microscope measurements.

**Figure 14:**
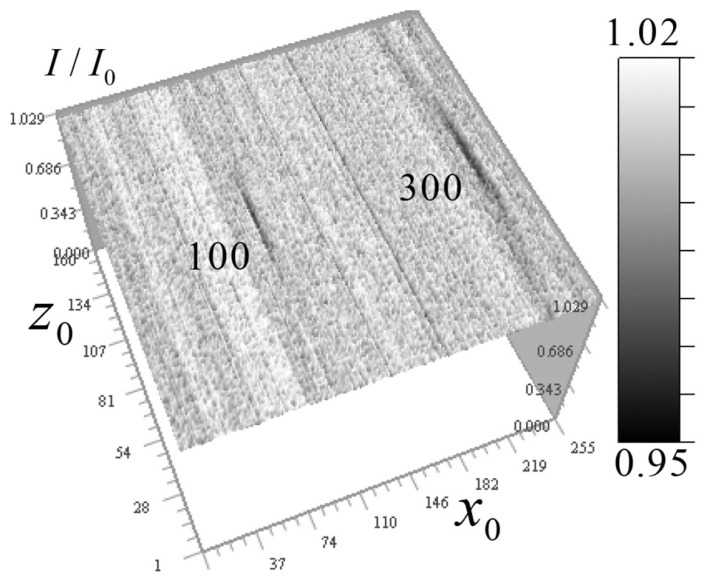
Measured signal distribution from gold balls with 100 and 300 nm diameters in the vertical plane 256 × 160 pixels (12.8 × 8 μm^2^) at *y*_0_ = 108 (5.4 μm).

Figure 14 shows the signal distribution of 100 and 300 nm gold balls in the vertical (along the lens optical axis) plane.

It can be seen that the axial blur of the measured signal is approximately 20–30 pixels, corresponding to 1–1.5 μm, depending on the ball size, likely due to 3-mirror objective aberrations caused by a residual axisymmetric error in the third mirror shape. Different areas of the mirror focus rays onto an optical axis segment rather than a single point.

**Figure 15:**
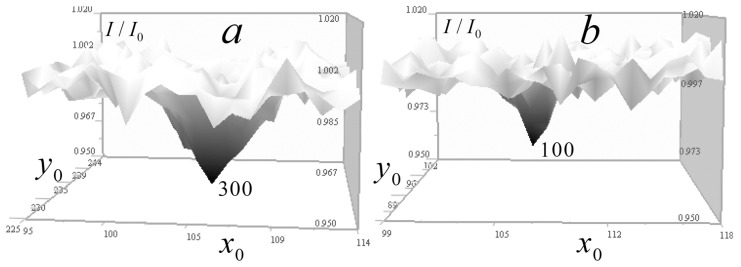
Measured signal distribution from gold balls with of 300 nm (**a**) and 100 nm (**b**) diameters on the horizontal plane 20 × 20 pixels (1 × 1 µm^2^).

Figure 15 shows the signal distributions from the balls at a reduced scale.

It is seen in Figure 14 that the response length in the vertical section significantly exceeds the horizontal plane response. Due to difficulty in describing such strong response in *z*_0_-direction, the kernel of the 3D tomography equation was determined from test objects from the above measurement data of gold balls. We assume that for a non-absorbing medium, this equation is a three-dimensional convolution similar to (4):

(14) $$\delta I=1-\frac{I}{I_{0}}(x_{0},y_{0},z_{0})=\int_{0}^{d}\iint \mu (x,y,z)K(x_{0}-x,y_{0}-y,z_{0}-z)dxdydz,$$

and the Fourier transform reduces the problem to solving a simple equation in *k*-space:

(15) $$\delta I(k_{x},k_{y},k_{z})=8\pi^{3}\mu (k_{x},k_{y},k_{z})K(k_{x},k_{y},k_{z}),$$

After calculating the spatial spectra $\delta I(k_{x},k_{y},k_{z})$ and $\mu (k_{x},k_{y},k_{z})$, the spatial spectrum of the desired kernel of the tomography equation is easily determined as $K(k_{x},k_{y},k_{z})=\delta I(k_{x},k_{y},k_{z})/8\pi^{3}\mu (k_{x},k_{y},k_{z})$. However, since the spectrum has near-zero components, this problem was solved with only minor Tikhonov regularization.

(16) $$K(k_{x},k_{y},k_{z})=\frac{\delta I(k_{x},k_{y},k_{z})\mu *(k_{x},k_{y},k_{z})}{8\pi^{3}\left|\mu (k_{x},k_{y},k_{z})\right|+\alpha_{k}},$$

where * denotes complex conjugation, and $\alpha_{k}$ is the regularization parameter.

**Figure 16:**
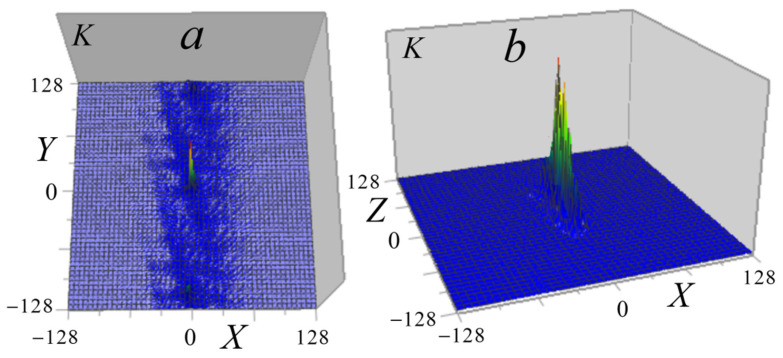
The kernel of the tomography equation $K(X=x_{0}-x,Y=y_{0}-y,Z=z_{0}-z)$: (**a**) the horizontal *XY* section at *Z* = 0; (**b**) the vertical *XZ* section at *Y* = 0.

Figure 16 shows the kernel of Equation (14) obtained by the inverse Fourier transform (16) in Cartesian coordinates.

As seen in Figure 16b, the axial *XZ* distribution of the experimental kernel differs significantly from the corresponding geometric-optical kernel of the ×46 microscope (see Figure 3b).

The resulting kernel was first tested and optimized based on the reconstruction of the absorption coefficient distribution of gold balls, using the real measured signal distribution with noise seen around the response from balls in Figure 14 and Figure 15. First, the problem of reconstructing the absorption coefficient *k*-spectrum from the *k*-spectrum of the initial signal distribution δI from gold beads was solved. However, as seen from Figure 15, there is a high-frequency random noise in the signal; therefore, this problem was also solved as ill-posed with Tikhonov regularization:

(17) $$\mu (k_{x},k_{y},k_{z})=\frac{\delta I(k_{x},k_{y},k_{z})K*(k_{x},k_{y},k_{z})}{\left|K(k_{x},k_{y},k_{z})\right|+\alpha [{\left(1+k_{x}^{2}+k_{y}^{2}+k_{x}^{2}\right)}^{2}]}.$$

**Figure 17:**
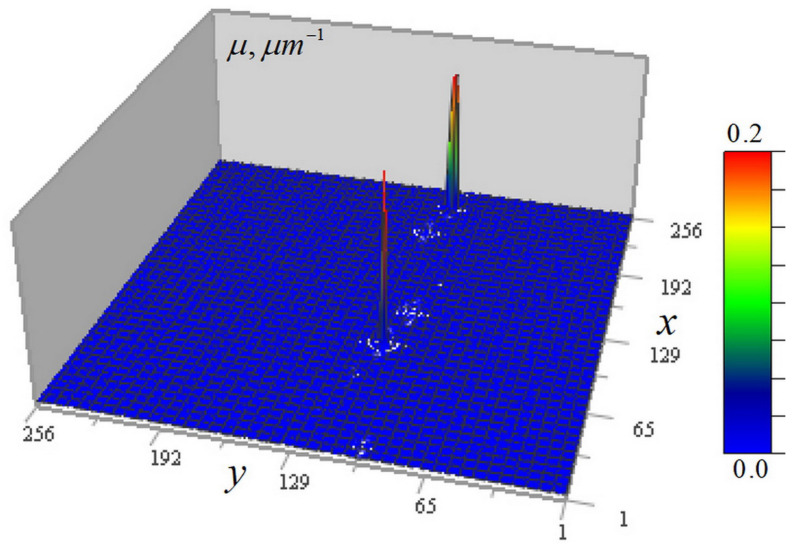
Reconstruction of the absorption coefficient of 100 and 300 nm gold balls. Tomographic cross section in the horizontal *xy* plane in region of 236 × 256 pixels (12.8 × 12.8 μm^2^) at *z* = 85 (4.25 μm).

**Figure 18:**
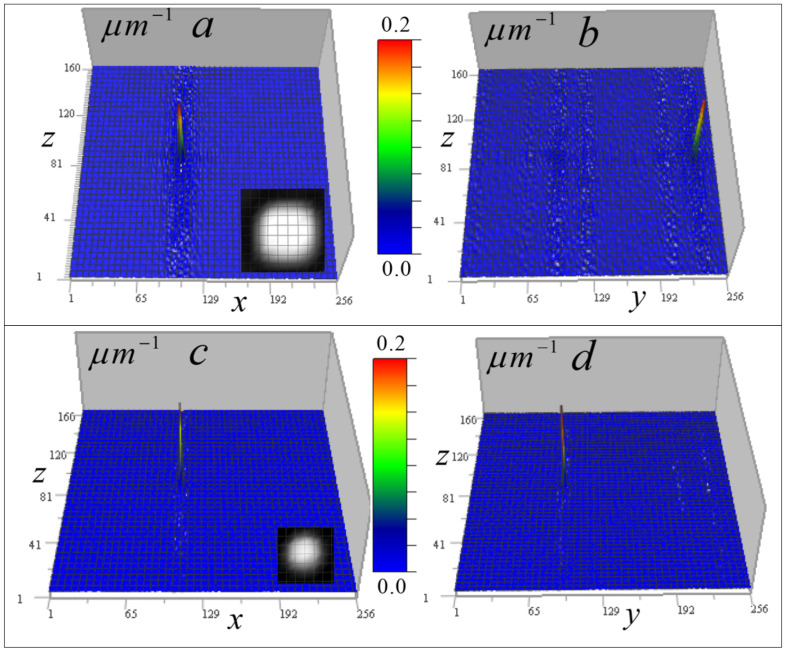
Reconstruction of the absorption coefficient of gold balls in vertical sections of 256 × 160 pixels (12.8 × 8 µm^2^): (**a**,**b**) 300 nm ball; (**c**,**d**) 100 nm ball; (**a**,**c**) in the *xz* planes; (**b**,**d**) in *yz* planes. Inserts show enlarged 2D balls’ reconstructions on these planes in grayscale.

Inverse Fourier transform determined the desired distribution μ(x,y,z) of the balls in Cartesian coordinates. The regularization coefficients in both (16) and (17) were optimized by the best match of the reconstructed absorption coefficient of the beads with the known gold absorption coefficient. Reconstruction results are shown in Figure 17 and Figure 18.

The arrangement of the balls on a transparent surface made it possible to show their reconstruction on a single horizontal section.

Figure 18 shows their tomographic sections in vertical planes.

The results show that the tomograms in both horizontal and vertical sections reproduce the gold absorption coefficient with 3–6% accuracy. Additionally, the enlarged images demonstrate that the shape and size of the balls are reproduced almost perfectly. Random errors are significantly reduced, largely due to error information inclusion in the kernel used in the reconstruction.

#### 3.2.2. Numerical Simulation

**Figure 19:**
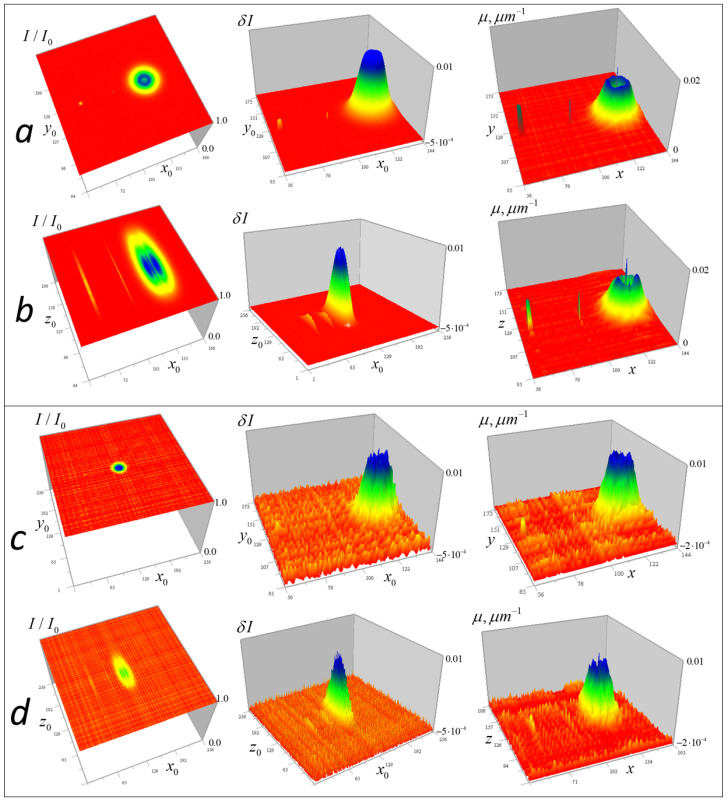
Numerical simulation of tomographic reconstruction using the experimental kernel of Equation (14): (**a**,**b**) reconstructions obtained with a simulated 1% random error level from the maximum of inhomogeneity based on Gaussian functions; (**c**,**d**) at 15% errors. (**a**,**c**) Horizontal sections; (**b**,**d**) vertical sections. Dimensions of the grid in the figures: (**a**) 127 × 127, 89 × 89, 89 × 89, (**b**) 127 × 127, 256 × 256, 89 × 89, (**c**) 256 × 256, 89 × 89, 89 × 89, (**d**) 256 × 256, 256 × 256, 127 × 127 pixels.

To study the algorithm resolution with the experimental kernel depending on the random error level, numerical simulation was performed and is demonstrated in Figure 19, similar to the simulation shown in Figure 5.

The left column in Figure 19 shows the received signal distributions with added random errors; the central column presents the corresponding distributions of the left side of the tomography of Equation (14), which display the contribution of the test objects to the signal. The right column demonstrates the tomographic distributions of the absorption coefficient of the test objects, reconstructed from these data using Equation (17) with regularization parameters *α* = 10^−9^ (Figure 19a,b) and *α* = 10^−8^ (Figure 19c,d).

In Figure 19b,d, stripes from small objects are visible on distributions of signal parameters, similar to gold ball traces in Figure 5. Nevertheless, despite the signal stretching in the z-direction, the shapes of the cubic and Gaussian inhomogeneities in vertical sections are reconstructed quite accurately even at high levels of random errors. Even at 15% error, the algorithm reconstructs 1-pixel inhomogeneities, but in Figure 19d, above and below the cubic and single-pixel inhomogeneities, one can notice duplicating artifacts of smaller size.

The error of the absorption coefficient reconstruction with 1% random error (Figure 19a,b) for Gaussian inhomogeneity is not more than 3.5% of its maximum value. For the cubic and separate 1-px inhomogeneities, error is 12%; for a 1-px inhomogeneity in a Gaussian cavity, it increases to 25%. With an error of 15%, increased regularization was applied in the reconstruction; its smoothing effect suppresses random errors but simultaneously reduces the magnitude of the reconstructed absorption inhomogeneities: the Gaussian inhomogeneity decreases by about 25–30%. Small inhomogeneities, although distinguishable, are smoothed much more than a large Gaussian inhomogeneity, by a factor of 3–4, because their spatial *k*-spectrum is closer to the spectrum of random error.

#### 3.2.3. Cell Tomography with ×345 EUV Microscope

**Figure 20:**
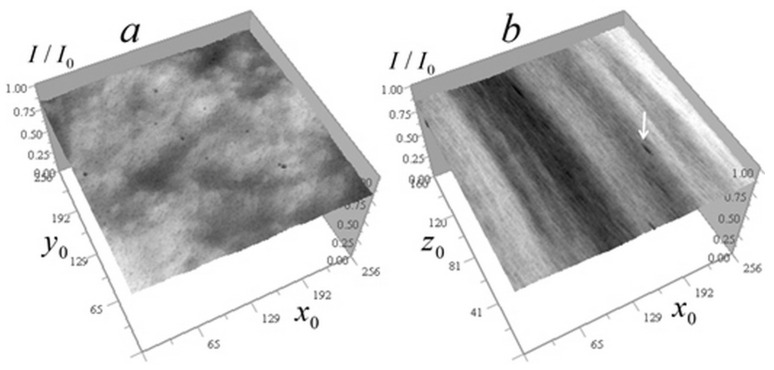
Measured signal distribution for a *Convallaria* cell: (**a**) in the horizontal plane $x_{0}y_{0}$ 256 × 256 pixels (12.8 × 12.8 µm^2^) at $z_{0}$ = 81 (4 µm); (**b**) in the vertical plane $x_{0}z_{0}$ 236 × 160 pixels (12.8 × 8 µm^2^) at $y_{0}$ = 81. Arrow indicates the trace of strong absorption inhomogeneity.

The experimental kernel was used to reconstruct the absorption coefficient of cells from ×345 EUV microscope measurements, where the field of view and analysis areas are correspondingly smaller than at ×46 magnification. The higher magnification in these measurements led to somewhat higher levels of random errors, particularly in vertical sections. Figure 20 shows the measured signal distributions for a *Convallaria* cell in two sections.

Figure 20 shows that the analyzed area of the cell is quite transparent, which confirms the applicability of algorithms (14)–(17). In Figure 20b, one can also see dark vertical traces left by inhomogeneities with relatively strong absorption. The one used in subsequent analysis is marked with a white arrow. Figure 20b also shows that it is easy to distinguish homogeneous z-layers in the vertical signal distribution, which enables the use of algorithms (9)–(13) in the future, after proper modification accounting for the shading cone seen in Figure 2 (right).

**Figure 21:**
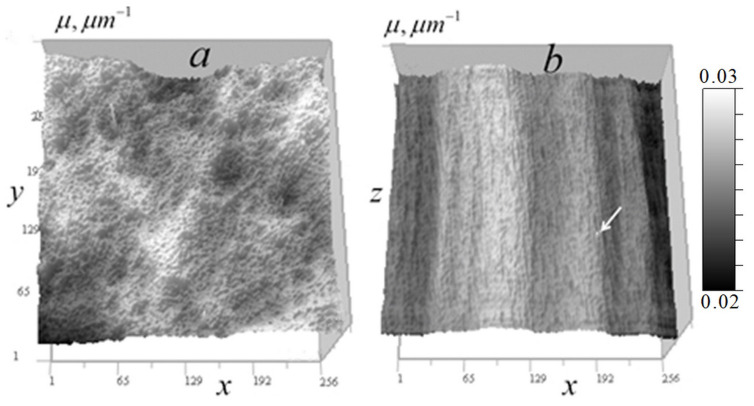
Reconstruction of the absorption coefficient distribution in a *Convallaria* cell. Tomographic sections: (**a**) in the horizontal *xy* plane 256 × 256 pixels (12.8 × 12.8 µm^2^) at *z* = 81 (4 µm); (**b**) in the vertical *xz* plane 256 × 160 pixels (12.8 × 8 µm^2^) at *y* = 128 (8 µm). The arrow indicates the reconstructed inhomogeneity responsible for the trace shown in Figure 20b.

Figure 21 displays the reconstructed absorption coefficient distribution in horizontal and vertical sections through the cell center, with the regularization parameter *a* = 10^−10^.

**Figure 22:**
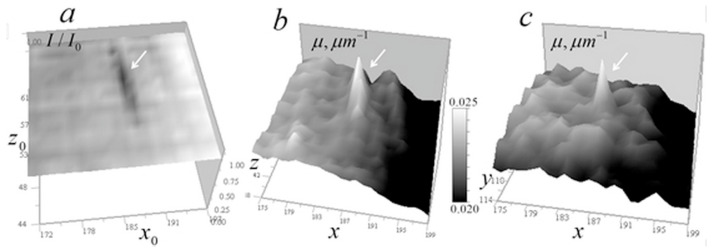
The reconstruction of a localized absorbing object: (**a**) enlarged object trace in the signal distribution in the vertical plane, marked in Figure 20b; (**b**) enlarged reconstruction of the object in the vertical *xz* plane at *y*_0_ = 128, with a size of 25 × 25 pixels (1.25 × 1.25 μm); (**c**) same object in the *xy* plane, size of 25 × 25 pixels (1.25 × 1.25 μm) at *z*_0_ = 55 (2.75 μm).

The horizontal tomogram (Figure 21a) reveals the multiscale structure of the cell. The vertical reconstruction (Figure 21b) demonstrates that this structure is highly correlated in the vertical direction. The object corresponding to the marked trace in Figure 20b is highly localized, as shown in this figure. A detailed reconstruction of this object is presented in Figure 22.

Figure 22b,c show that the reconstruction algorithm strongly localizes the object that generated the stretched signal observed in Figure 22a. Figure 22c suggests that this may be the core of an annular (spheroidal) formation. Its linear size in the horizontal plane is 2 pixels (100 nm), while in the vertical plane, it is approximately 150 nm. As shown in the simulation (Figure 19d), this vertical elongation may be caused by high noise levels.

**Figure 23:**
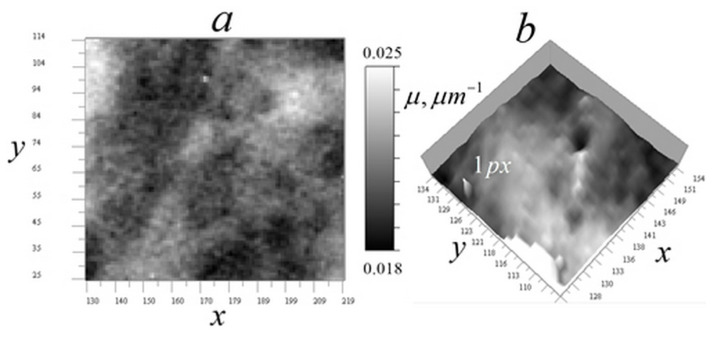
Small inhomogeneities observed in tomograms of the cell: (**a**) ring structures in the *xy* region of 90 × 90 pixels (4.5 × 4.5 µm^2^); (**b**) single-pixel natural inhomogeneities and an embedded 1-pixel model inhomogeneity in the *xy* region of 30 × 30 pixels (1.5 × 1.5 µm^2^).

In Figure 23, tomograms of small inhomogeneities not visible in vertical signal distributions due to high noise levels are shown.

In Figure 23a, there are numerous ring inhomogeneities visible in a relative-transparency region. These are similar to those found in another *Convallaria* cell (Figure 10b), presumably sections of smaller, and more transparent spheroidal bodies. Their diameter is approximately 100–350 nm. Figure 23b shows a horizontal tomogram cell section with the smallest 1-pixel inhomogeneities along with an artificial 1-pixel inhomogeneity inserted into the tomogram for comparison. This result confirms the 50 nm resolution of the ×345 microscope.

**Figure 24:**
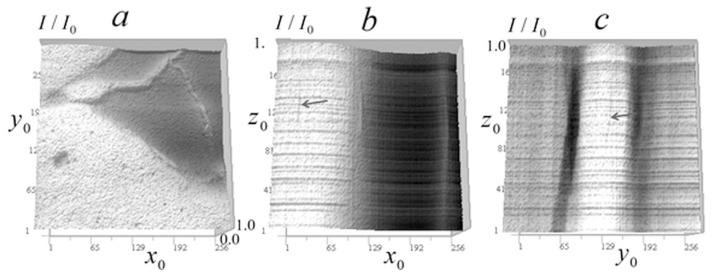
Measured signal distribution in neurites: (**a**) in the horizontal plane of 256 × 256 pixels (12.8 × 12.8 μm^2^) at *z* = 100 (5 μm); (**b**) in the vertical plane of 256 × 160 pixels (12.8 × 8 μm^2^) at *y* = 140 (7 μm); (**c**) in the vertical plane 256 × 160 (12.8 × 8 μm^2^) at *x* = 3 5 (1.8 μm). Arrow indicates the trace of strong absorption inhomogeneity.

In Figure 24, tomographic measurements for another cell (neurites of mouse cerebellar granule cells) is presented.

It can be seen from the signal distributions in Figure 24 that there are areas of relative transparency and areas with significant attenuation of the signal within the cell. On the vertical sections of Figure 24b,c, only a few significant inhomogeneities are observed, leaving vertical traces similar to those in Figure 14 and Figure 21.

**Figure 25:**
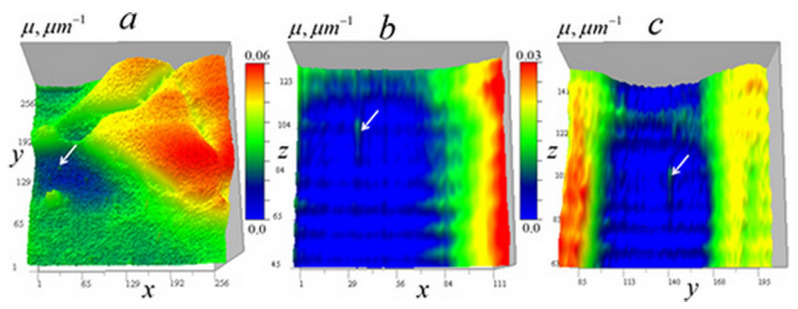
Reconstruction of the absorption coefficient in the neurites of cerebellar granule cell in tomography sections: (**a**) in the horizontal *xy* plane 256 × 256 pixels (12.8 × 12.8 μm^2^) at *z* = 100 (5 μm); (**b**) in the vertical *xz* plane of 112 × 78 pixels (5.6 × 3.9 μm^2^) at *y* = 140 (7 μm); and (**c**) in the vertical *yz* plane of 112 × 78 pixels (5.6 × 3.9 μm^2^) at *x* = 35 (1.8 μm). The inhomogeneities that left traces in the signal in Figure 24b,c marked with arrows.

**Figure 26:**
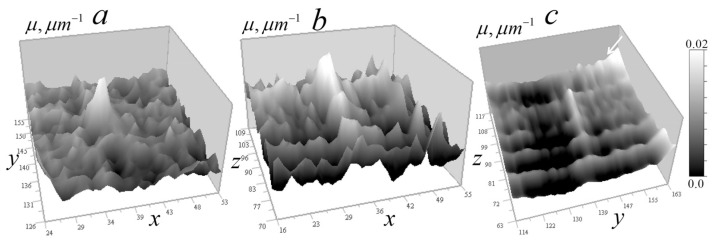
Enlarged reconstruction of the absorption coefficient for small object in neurites of cerebellar granule cell. Tomographic sections: (**a**) in the horizontal *xy* plane of 30 × 30 pixels (1.5 × 1.5 μm^2^; (**b**) the vertical *xz* plane of 40 × 40 pixels (2 × 2 μm^2^); (**c**) the vertical *yz* plane of 50 × 50 pixels (2.5 × 2.5 μm^2^).

Figure 25 and Figure 26 present the reconstruction of the absorption coefficient for the object whose trace is indicated by arrows in Figure 24b,c with the regularization parameter *a* = 10^−10^.

In the horizontal section in Figure 25a, the arrow indicates the reconstructed inhomogeneity in the absorption coefficient of the object that produced stretched traces in the vertical sections in Figure 24b,c. In the vertical sections in Figure 25b,c, this stretched signal distribution is reconstructed into a localized inhomogeneity in the absorption coefficient at the level z_0_ = 100 (5 µm). These reconstructions are shown on an enlarged scale in Figure 26.

The horizontal section in Figure 26a reveals fine structural details of inhomogeneity. This inhomogeneity has an absorption value 2–3 times larger than that discussed in Figure 20, Figure 21 and Figure 22, with a lateral size of 2–3 pixels (100–150 nm). The vertical sections in Figure 26b,c show that the reconstructed inhomogeneity exhibits greater spread in the z-direction compared to the object in Figure 22b. Similar to Figure 19d, small peaks appear above and below the *z*-axis, which may be artifacts. However, they are rather related to the variations in the signal between the z-levels observed in Figure 24b,c due to its instability to random errors, the level of which they exceed by 2–3 times.

**Figure 27:**
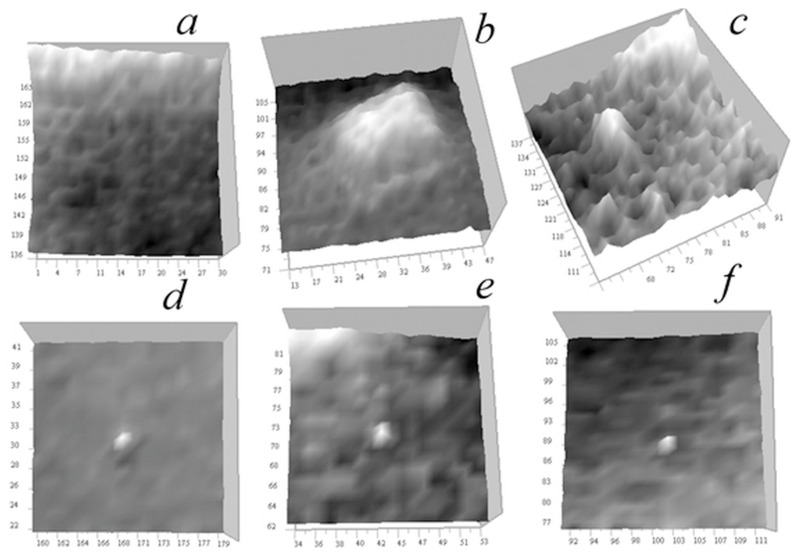
Reconstruction of the absorption coefficient in various areas of the neurites of cerebellar granule cell in horizontal sections: (**a**–**c**) in areas with dimensions of 30 × 30 (1.5 × 1.5 μm^2^); (**d**–**f**) in areas with dimensions of 20 × 20 (1 × 1 μm^2^).

Figure 27 shows tomograms of inhomogeneities observed in various regions of the neurites of a cerebellar granule cell in the horizontal section, including small single-pixel inhomogeneities that are not visible in vertical signal distributions (Figure 25b,c).

In Figure 27a, one can see ring structures, presumably shells of spheroidal bodies, similar to those found in *Convallaria* cells (Figure 8, Figure 12, Figure 13 and Figure 23), although they are atypical for this cell. Their linear size is 150–300 nm, and the shell thickness is 50–200 nm. The absorption contrast with the environment is 0.0005–0.003 μm^−1^. Figure 27b,c show large organelles against a complex relief.

Single small objects with sizes less than 100 nm are shown in Figure 27d–f. In Figure 27d, the object is located in an almost homogeneous translucent medium; in Figure 27e, the object is shown surrounded by other less contrasting inhomogeneities with sizes from 50 to 150 nm; Figure 27f shows the object surrounded by ring-shaped structures.

The presented results demonstrate that the ×345 EUV microscope can detect and reconstruct individual small inhomogeneities with lateral dimensions as small as 50 nm, though their axial extent is elongated to approximately 150 nm in the reconstruction. Numerical simulations suggest that the resolution along the optical axis could be significantly improved by minimizing measurement errors.

## 4. Discussion

The proposed axial tomography method based on all-reflective SX microscopes offers new possibilities for studying biological cells without organelle staining used in optical diagnostics. This avoids the modifications caused by staining agents, which can alter cellular structures and result in loss of information about the biological environment.

The results presented in X-ray nanotomography confirm the promising nature of the method and suggest further improvement. The authors are currently developing a mirror-based SR microscope at the 3.37 nm wavelength (in water transparency window), enabling both cryo-preservation of hydrated samples and live observation of unfrozen hydrated cells in vacuum-sealed cells.

On the theoretical side, the authors plan to continue research by determining the kernel of the tomography equation from measurements of known test objects and incorporating this kernel into the geometric optical method presented in this article for inhomogeneities in absorbing media. This approach could significantly expand the method’s applicability.

## 5. Conclusions

This article presents the results of developing the theory, methods, and algorithms of axial nanotomography using soft X-ray microscopes with 46- and 345-fold magnification. A generalization of the geometric-optical theory of probing radiation formation for inhomogeneities of the absorption coefficient in an absorbing medium has been performed, and the 3D convolution equation for the tomography inverse problem has been obtained, which significantly expands the field of diagnostic applicability. A novel reconstruction algorithm for quantitative analysis (metrology) of absorbing cells was developed, tested in numerical simulations, and applied to study *Convallaria* plant cells using the ×46 EUV microscope. The results confirm a 140 nm resolution capability in analyzing thin structures in elevated-absorption cell regions that were previously unresolvable using the weak absorption algorithm [14].

For the ×345 EUV microscope, another tomography method was developed, in which the kernel of a 3D convolution-type equation was determined from an experiment with test objects with a known absorption coefficient, shape, and position. Testing of the algorithm of this method in numerical modeling and the results of its application in the analysis of plant cells of *Convallaria* and in cells of neurite grains of mouse cerebellum demonstrated its ability to diagnose small single organelles with a size of 50 nm.

The developed tomography does not require staining of organelles, which is used in optical microscopy, or cutting cells into very thin slices, as is done in electron microscopy. The results obtained open up new possibilities for X-ray nanotomography in quantitative analysis of cell structure. This is undoubtedly of interest to biologists and in other fields where these diagnostic methods are applicable. The presented methods have significant potential for future development.

## Figures and Tables

**Table 1:** Key experimental parameters for cell reconstructions with the two EUV microscopes.

	Grid Dimension	Pixel Size	Noise Level	Regularization
×46 microscope	256 × 256 × 160 (213)	140 nm	0.002–0.005	0
×345 microscope	256 × 256 × 160	50 nm	0.002–0.005	*α* = 10^−8^–10^−10^

## Data Availability

The raw data supporting the conclusions of this article will be made available by the authors on request.

## Abbreviations

The following abbreviations are used in this manuscript:

IPM RAS	Institute for Physics of Microstructures, Russian Academy of Sciences
EUV	Extremal ultraviolet wavelength range
SX-microscopy	Soft X-Ray microscopy
STED-microscopy	Stimulated emission depletion microscopy

## References

[1] Fogelqvist E., Kördel M., Carannante V., Önfelt B., Hertz H.M. (2017). Laboratory cryo x-ray microscopy for 3D cell imaging. Sci. Rep..

[2] Dehlinger A., Seim C., Stiel H., Twamley S., Ludwig A., Kördel M., Grötzsch D., Rehbein S., Kanngießer B. (2020). Laboratory soft X-ray microscopy with an integrated visible-light microscope—Correlative workflow for faster 3D cell imaging. Microsc. Microanal..

[3] Groen J., Conesa J.J., Valcarcel R., Pereiro E. (2019). The cellular landscape by cryo soft X-ray tomography. Biophys. Rev..

[4] Weinhardt V., Chen J.-H., Ekman A., McDermott G., Le Gros M.A., Larabell C. (2019). Imaging cell morphology and physiology using X-rays. Biochem. Soc. Trans..

[5] Torrisi A., Wachulak P., Węgrzyński Ł., Fok T., Bartnik A., Parkman T., Vondrová Š., Turňová J., Jankiewicz B.J., Bartosewicz B. (2017). A stand-alone compact EUV microscope based on gas-puff target source. J. Microsc..

[6] Ejima T., Ishida F., Murata H., Toyoda M., Harada T., Tsuru T., Hatano T., Yanagihara M., Yamamoto M., Mizutani H. (2010). High throughput and wide field of view EUV microscope for blur-free one-shot imaging of living organisms. Opt. Express.

[7] Wachulak P.W., Torrisi A., Bartnik A., Wegrzynski Ł., Fok T., Fiedorowicz H. (2017). A desktop extreme ultraviolet microscope based on a compact laser-plasma light source. Appl. Phys. B.

[8] Sage D., Donati L., Soulez F., Fortun D., Schmit G., Seitz A., Guiet R., Vonesch C., Unser M. (2017). DeconvolutionLab2: An open-source software for deconvolution microscopy. Methods.

[9] Yang X., Yang Z., He Y. (2020). Mitochondrial dynamics quantitatively revealed by STED nanoscopy with an enhanced squaraine variant probe. Nat. Commun..

[10] Leigh K.E., Navarro P.P., Scaramuzza S., Chen W., Zhang Y., Castaño-Díez D., Kudryashev M. (2019). Subtomogram averaging from cryo-electron tomograms. Methods Cell Biol..

[11] Utke I., Moshkalev S., Russell P. (2012). Nanofabrication Using Focused Ion and Electron Beams: Principles and Applications. Oxford Series on Nanomanufacturing.

[12] Malyshev I.V., Reunov D.G., Chkhalo N.I. (2022). High-aperture EUV microscope using multilayer mirrors and a 3D reconstruction algorithm based on z-tomography. Opt. Express.

[13] Gaikovich K.P., Malyshev I.V., Reunov D.G., Chkhalo N.I. (2023). The theory of axial tomography based on the inverse Radon transform for high-aperture soft X-ray microscopy. Tech. Phys..

[14] Gaikovich K.P., Malyshev I.V., Reunov D.G., Chkhalo N.I. (2024). Investigations of Microscopic X-ray tomography. Tech. Phys..

[15] Gaikovich K.P., Gaikovich P.K., Maksimovitch Y.S., Badeev V.A. (2012). Pseudopulse Near-Field Subsurface Tomography. Phys. Rev. Lett..

[16] Radon J. (1917). Uber Die Bestimmung von Funktionen Durch Ihre Integralwerte Langs Gewisser Mannigfaltigkiten.

[17] Tikhonov A.N. (1977). Solution of Ill-Posed Problems.

